# From Pathogenesis to Treatment—New Perspectives in Rheumatology

**DOI:** 10.3390/ijms242115590

**Published:** 2023-10-26

**Authors:** Elena Rezus, Catalin Codreanu

**Affiliations:** 1 Department of Rheumatology, “Grigore T Popa” University of Medicine and Pharmacy, 700115 Iasi, Romania; 2 Center for Rheumatic Diseases, “Carol Davila” University of Medicine and Pharmacy, 050472 Bucharest, Romania

Rheumatic diseases are characterized by complex pathogenic mechanisms, with intricate signaling pathways and various imbalances of proinflammatory and anti-inflammatory cytokines, especially in the case of immune-inflammatory conditions. A deep knowledge of these mechanisms is required in order to attain the best possible outcomes through treatment. Elements such as biomarkers are essential in evaluating disease progression and are a hallmark of potential disease-related damage. Knowledge of such biomarkers would be of immeasurable help in preventing disease complications, allowing for physicians to be one step ahead and implement damage control. In sum, pathogenesis and its markers directly influence the choice of treatment strategy.

The new Special Issue titled “From Pathogenesis to Treatment—New Perspectives in Rheumatology” of the International Journal of Molecular Sciences encompasses a total of eight contributions: three original articles and five reviews, presenting novel information regarding pathogenic mechanisms, biomarkers, and therapy of diseases such as rheumatoid arthritis, axial spondylarthritis, systemic lupus erythematosus, systemic sclerosis, adult-onset Still’s disease, and eosinophilic fasciitis.

In a nested prospective observational case–control study, Mena-Vázquez et al. [1] analyzed various potential biomarkers that may change the approach to the diagnosis and follow-up of rheumatoid arthritis-interstitial lung disease (RA-ILD) and lessen the respiratory burden of rheumatoid arthritis (RA). The authors evaluated the cytokine and inflammation levels in patients diagnosed with RA-ILD compared to patients with RA, but not interstitial lung disease (ILD). The study found higher disease activity, according to the Disease Activity Score with Erythrocyte Sedimentation Rate (DAS28-ESR), to be a constant in patients with RA-ILD, as well as higher titers of anti-citrullinated peptide antibodies (ACPA). This data are in line with results from various other studies [1]. Several cytokines and chemokines, such monocyte chemoattractant protein 1 (MCP-1), also known as C-C motif ligand 2 (CCL2), stromal cell-derived factor 1 alpha (SDF-1 alpha), and interleukin-18 (IL-18), were investigated for their correlation with RA-ILD.

In a study that involved 78 RA patients, Dinache et al. [2] investigated the correlation between the disease characteristics in patients diagnosed with ILD and radiological features, observed on both chest X-rays and high-resolution computer tomography (HR-CT). Chest radiograph elements were found to differ according to gender, rheumatoid factor, and ACPA titers, while the HR-CT findings were also correlated with ACPA titer and different therapeutic agents. The study underlines the necessity for thorough clinical evaluation, as symptoms are nonspecific and, quite often, of a subtle nature.

One of the autoimmune diseases with the highest mortality is systemic sclerosis. A proper assessment on the topic of the cellular and molecular mechanisms involved in its pathogenesis is essential in truly understanding the disease. Bratoiu et al. [3] elaborated a comprehensive review on the subject of muscle involvement in systemic sclerosis, highlighting the clinical outcomes for each of the pathological mechanisms described. Regarding vascular smooth muscles and how they are affected in systemic sclerosis, the importance of endothelial-to-mesenchymal transition (EndoMT) was underlined, given its influence on the microvasculature and fibrosis, both in the skin and internal organs. Another issue that was investigated by this study was the transition of vascular smooth muscle cells (VSMCs), a contributing factor in the development of fibrosis and pulmonary hypertension. In terms of esophageal involvement, the authors analyzed various studies on systemic sclerosis patients with esophageal dysmotility, with an emphasis on molecular and histological aspects. Several studies regarding the histological involvement of the gastric wall were evaluated.

The review covers the clinical and serological features of skeletal myopathy and, additionally, muscle biopsy patterns. Another area of interest is represented by the concise summary of multiple studies regarding the presence of PM/Scl antibodies in patients with systemic sclerosis, including patients from the EUSTAR database. The review broached the topic of scleromyositis and the presence of different antibody profiles that may classify this overlap syndrome into different subsets. One major factor that influences mortality in systemic sclerosis is cardiovascular involvement. The authors addressed the importance of CMR (cardiac magnetic resonance) in the diagnosis of cardiac manifestations of systemic sclerosis, extensively presenting the most recent studies regarding this subject.

Fibrosis of the myocardium and the conductive system is the cause of arrythmias and conduction defects. Routine screening is needed in order to more readily diagnose and treat patients. This matter was elaborately reviewed by Vrancianu et al. [4], with the aim of exploring the risk factors and characteristics of patients that present these manifestations, in order to lower mortality by implementing treatment in the subclinical stage. Supraventricular premature beats (SPB), premature ventricular contractions (PVC), and right bundle branch block (RBBB) were the most prevalent supraventricular and ventricular arrythmias and conduction defects, respectively. Serological findings, such as high levels of N-terminal prohormones of brain natriuretic peptides (NT-proBNP), high-sensitivity troponin I (hs-TnI), and high-sensitivity troponin T (hs-TnT) values, were associated with supraventricular and ventricular arrythmias, while hs-TnT was correlated with RBBB. Regarding Holter monitoring, a lower turbulence slope (TS) value proved to be an independent predictor for ventricular arrythmias.

Systemic lupus erythematosus (SLE) is a complex pathology that involves intricate mechanisms and the contribution of both the innate and adaptive immune system. One of the critical pathogenetic factors is represented by the interferons (IFNs) pathway. IFNs can activate the Janus kinase/signal transducers and activators of transcription (JAK/STAT) pathway, and, thus trigger the immune response. The review by Richter et al. [5]. addressed a new therapeutic perspective in SLE by evaluating the potential role of Janus kinase (JAK) inhibitors. The review focused on the performances of Tofacitinib and Baricitinib in SLE, but also delved into the effects of newer JAK inhibitors, highlighting ongoing studies. Tofacitinib has demonstrated positive effects in murine SLE models in areas such as serology, cutaneous involvement, and proteinuria. One of the important aspects regarding Baricitinib treatment in SLE is the improvement of renal involvement in a murine model, leading to the recovery of structural proteins in podocytes.

The importance of disease progression is further emphasized in a study by Favero et al. [6] regarding early axial spondyloarthritis. A serum biomarker, fetuin-A, a plasma carrier protein for the calcium and phosphate produced in the liver, was found to be associated with radiographic damage of the sacroiliac joints. Fetuin-A has multiple roles, including involvement in bone metabolism and inflammation. The study suggests a potential role for fetuin-A as a predictor for structural damage, with a sensitivity that may surpass that of C-reactive protein (CRP).

The treatment of rare diseases is challenging, more so when diagnoses of said diseases are difficult to establish. Adult-onset Still’s disease is an inflammatory pathology with severe complications and requires the exclusion of other, much more prevalent pathologic entities such as infection, neoplasia, and other immune diseases. A complete tableau of the pathology, from its clinical characteristics to treatment, was presented by Macovei et al. [7], emphasizing therapy categories, especially in patients with an unfavorable disease progression under glucocorticoids and conventional synthetic disease-modifying antirheumatic drugs (csDMARDs). IL-1 and IL-6 blockade treatment was addressed, with a few studies assessing their efficacy, but also other therapeutic options, including IL-18, tumor necrosis factor- α (TNF-α), IL-17, and JAK inhibitors.

While diseases with a complex pathogenic mechanism represent a challenge, poorly understood pathogeny is truly an obstacle, especially in terms of treatment. Such is the case with eosinophilic fasciitis, evaluated in an elaborate review by Mazilu et al. [8]. The authors sought to address subjects such as etiology, known pathogenesis, histological findings, investigations, diagnosis, and treatment. Its differential diagnosis was exhaustively reviewed, including other rare diseases, for instance, systemic sclerosis, nephrogenic systemic fibrosis, eosinophilia–myalgia syndrome, and toxic oil syndrome, among others. Given that no randomized studies have been carried out examining therapy for eosinophilic fasciitis, the presented treatment options are based on medical experience, case reports, and series of cases. The evaluated agents comprise GC, csDMARDs (methotrexate, sulfasalazine, and azathioprine), biological disease-modifying antirheumatic drugs (bDMARDs), such as infliximab, rituximab, and tocilizumab, and targeted synthetic disease-modifying antirheumatic drugs (tsDMARDs), such as Baricitinib.

The majority of the articles included in this Special Issue comprise reviews of the literature. Further studies are needed for each of the topics presented, especially given the direct effect this research can have on implementing diagnosis tools, elements of screening and predicting disease progression, and, ultimately, treatment strategies. Given the novelty and thorough presentation of the subjects, the content of this Special Issue, in its totality, should be of particular interest to clinicians, as it endeavors to progressively unravel each topic, from more well-known elements, such as clinical aspects, to the pathogenesis of complex diseases, novel biomarkers, and therapeutic options, all essential to clinical practice.

## References

[1] Mena-Vázquez N., Godoy-Navarrete F.J., Lisbona-Montañez J.M., Redondo-Rodriguez R., Manrique-Arija S., Rioja J., Mucientes A., Ruiz-Limón P., Garcia-Studer A., Ortiz-Márquez F. (2023). Inflammatory Biomarkers in the Diagnosis and Prognosis of Rheumatoid Arthritis-Associated Interstitial Lung Disease. Int. J. Mol. Sci..

[2] Dinache G., Popescu C.C., Mogoșan C., Enache L., Agache M., Codreanu C. (2022). Lung Damage in Rheumatoid Arthritis-A Retrospective Study. Int. J. Mol. Sci..

[3] Bratoiu I., Burlui A.M., Cardoneanu A., Macovei L.A., Richter P., Rusu-Zota G., Rezus C., Badescu M.C., Szalontay A., Rezus E. (2022). The Involvement of Smooth Muscle, Striated Muscle, and the Myocardium in Scleroderma: A Review. Int. J. Mol. Sci..

[4] Vrancianu C.A., Gheorghiu A.M., Popa D.E., Chan J.S.K., Satti D.I., Lee Y.H.A., Hui J.M.H., Tse G., Ancuta I., Ciobanu A. (2022). Arrhythmias and Conduction Disturbances in Patients with Systemic Sclerosis-A Systematic Literature Review. Int. J. Mol. Sci..

[5] Richter P., Cardoneanu A., Burlui A.M., Macovei L.A., Bratoiu I., Buliga-Finis O.N., Rezus E. (2022). Why Do We Need JAK Inhibitors in Systemic Lupus Erythematosus?. Int. J. Mol. Sci..

[6] Favero M., Ometto F., Belluzzi E., Cozzi G., Scagnellato L., Oliviero F., Ruggieri P., Doria A., Lorenzin M., Ramonda R. (2023). Fetuin-A: A Novel Biomarker of Bone Damage in Early Axial Spondyloarthritis. Results of an Interim Analysis of the SPACE Study. Int. J. Mol. Sci..

[7] Macovei L.A., Burlui A., Bratoiu I., Rezus C., Cardoneanu A., Richter P., Szalontay A., Rezus E. (2022). Adult-Onset Still’s Disease-A Complex Disease, a Challenging Treatment. Int. J. Mol. Sci..

[8] Mazilu D., Boltașiu L.A., Mardale D.-A., Bijă M.S., Ismail S., Zanfir V., Negoi F., Balanescu A.R. (2023). Eosinophilic Fasciitis: Current and Remaining Challenges. Int. J. Mol. Sci..

